# The Role of the TG2-GPR56 Complex in Cutaneous Squamous Cell Carcinoma (CSCC) Aggression and Therapeutic Resistance

**DOI:** 10.3390/ijms27062902

**Published:** 2026-03-23

**Authors:** David J. Weber, Mary E. Cook, Wenbo Yu, Maximino Redondo, Raquel Godoy-Ruiz

**Affiliations:** 1 Department of Biochemistry and Molecular Biology, University of Maryland School of Medicine, Baltimore, MD 21201, USA; dweber@som.umaryland.edu (D.J.W.);; 2 Institute for Bioscience and Biotechnology Research (IBBR), University of Maryland, Rockville, MD 20850, USA; 3 The Center for Biomolecular Therapeutics (CBT), University of Maryland School of Medicine, Baltimore, MD 21201, USA; 4 Computer Aided Drug Design Center, Department of Pharmaceutical Sciences, School of Pharmacy, University of Maryland Baltimore, Baltimore, MD 21201, USA; 5 Department of Biochemistry, Molecular Biology and Immunology, School of Medicine, University of Malaga, 29010 Malaga, Spain; 6 Research Network on Chronicity, Primary Care and Prevention and Health Promotion (RICAPPS), Research and Innovation Unit, Costa del Sol University Hospital, 29603 Marbella, Spain; 7 School of Health Sciences and Sports, Alfonso X University Mare Nostrum, 29004 Malaga, Spain

**Keywords:** cutaneous squamous cell carcinoma (cSCC), epidermal cancer stem (ECS) cells, transglutaminase-2 (TG2), G-protein-coupled receptor GPR56, TG2-GPR56 complex

## Abstract

Cutaneous squamous cell carcinoma (cSCC) is the second most prevalent skin cancer diagnosed worldwide after basal cell carcinoma. CSCC represents a growing global public health challenge due to its higher potential of local invasion, recurrence, and metastasis. Incidence rates of cSCC are projected to increase due to rising exposures to risks factors. Ultraviolet light exposure is the primary cause, and lighter skin pigmentation, immunosuppressive conditions and skin phototype are the primary risk factors. CSCC typically presents as a red, scaly, flat lesion (in situ tumors) or a red, firm, raised lesion with scale or erosion (invasive tumors). Surgical excision remains the standard-of-care for localized cSCC and is often curative. Although, most patients achieve favorable outcomes, a subset of cSCC exhibits a highly aggressive and metastatic phenotype (postoperative recurrence rates are approximately 5%). Addressing the clinical challenge posed by these high-risk cases requires a more comprehensive understanding of the underlying molecular drivers. This review examines the interaction between transglutaminase 2 (TG2) and the G-protein-coupled receptor 56 (GPR56) as a pivotal driver of the aggressive cSCC phenotype. This molecular axis is particularly significant for its role in the maintenance of epidermal cancer stem (ECS) cells, which contribute to tumor progression and therapy resistance. While the definitive link between the TG2-GPR56 complex and systemic metastasis in cSCC is currently being elucidated, significant evidence from analogous malignancies and in vitro keratinocyte models provides a clear mechanistic roadmap for its involvement in tumor invasion.

## 1. Introduction

Cutaneous squamous cell carcinoma (cSCC), the second most common form of skin cancer worldwide, has become a serious public health concern, particularly in regions with high ultraviolet radiation (UVR) exposure such as North America, Australia, New Zealand, and Europe [1,2,3,4,5,6]. In the United States, the estimated annual incidence of cSCC alone surpasses one million cases [7]. The etiology of cSCC is multifactorial, driven by a complex interplay of environmental, genetic, immunologic and biological factors, with susceptibility heavily influenced by constitutional risks factors, such as light skin pigmentation [6]. Notably, beyond its role as a primary environmental carcinogen, UVR exerts broad homeostatic effects by engaging the skin’s highly organized neuro-immuno-endocrine system, allowing the organism to adapt to its sunlit environment [8,9]. Furthermore, different wavelengths of UVR penetrate the skin to varying depths, eliciting distinct biological effects. Specifically, ultraviolet B (UVB, 280 to 315 nm) is the primary environmental stressor driving the most common skin malignancies, basal cell and squamous cell carcinomas, whereas both UVB and ultraviolet A (UVA, 315 to 400 nm) radiations contribute to the development of cutaneous melanoma. Paradoxically, to protect against excessive inflammation following UV-induced cellular damage, UVR also triggers localized immune–endocrine responses, which create a profoundly immunosuppressive environment. This severely compromises immune surveillance, ultimately enhancing epidermal carcinogenesis and facilitating tumor progression [8].

Recent epidemiological data from European cancer registries indicate a steady escalation in disease burden, with annual incidence increasing between 2.4% and 5.7% from 2023 to 2024 [7]. This upward trend is reflected in global longitudinal analyses spanning 1990 to 2021. During this period, the prevalence of cSCC in individuals aged 15 to 95+ years increased by 345%, rising from approximately 510,851 cases (95% UI: 398,768–637,037) to over 2,275,834 cases (95% UI: 1,924,479–2,705,755) [6]. Over the same timeframe, the age-standardized prevalence rate (ASPR) increased by 201%, from 14.69 to 26.85 per 100,000 population. Likewise, the age-standardized incidence rate (ASIR) showed a 67% increase, growing from 13.38 (95% UI: 10.55–17.26) to 22.38 (95% UI: 19.90–25.27) over the same timeframe [6]. These figures underscore the rapidly intensifying impact of cSCC on global healthcare systems [6,7].

CSCC typically presents as a red, scaly, flat lesion (in situ tumors) or a red, firm, raised lesion with scale or erosion (invasive tumors). Most cSCC tumors originate from precursor lesions, such as actinic keratoses and squamous cell carcinoma in situ, through a process of accumulated genetic mutations. Despite the fact that most of cSCC cases are effectively managed via surgical excision and cured via local ablation or surgical excision with clear margins, a clinically significant proportion of tumors are considered unresectable or pose complex challenges [7,10]. This is often due to anatomic constraints where lesions reside near critical anatomical structures, making surgery likely to result in unacceptable functional loss or disfigurement, or due to advanced disease involving deep-tissue invasion [7,11]. The clinical management of this highly aggressive cSCC subset remains exceedingly complex, as these cases are intrinsically linked to an elevated risk of local recurrence, systemic metastasis, and disease-specific mortality [7].

A fundamental driver of this clinical progression is tumor cell plasticity, particularly the transition from epithelial to mesenchymal states (Epithelial–Mesenchymal Transition, or EMT) [12,13,14,15]. EMT enables polarized epithelial keratinocytes to undergo multiple epigenetic and phenotypic changes, losing their adhesion molecules and acquiring migratory properties that facilitate basement membrane degradation, intravasation, extravasation, and the development of chemoresistance [16,17]. Consequently, EMT serves as a critical driver of the metastatic cascade by facilitating tumor initiation and the acquisition of stemness, a state characterized by enhanced self-renewal, cellular plasticity and profound therapeutic resistance [17,18,19]. Within this framework, epidermal cancer stem (ECS) cells represent the primary subpopulation responsible for tumor maintenance, progression, and relapse [1,18,20]. ECS cells exhibit a highly aggressive phenotype, characterized by an inherent capacity for self-renewal, high-level expression of pluripotency markers, and profound resistance to conventional therapeutic interventions, which typically targets differentiated cancer cells while sparing the stem cell population [1]. Ultimately, these cells are the primary drivers behind the formation of rapidly proliferating, invasive, and highly vascularized tumors that are prone to recurrence and metastasis [21,22].

Current research identifies transglutaminase 2 (TG2) as a critical survival factor that is significantly upregulated within the ECS cell subpopulation, serving as a central driver of the aggressive cSCC phenotype [1,20,21,23,24,25,26]. Crucial for ECS cell maintenance, TG2 acts as a molecular scaffold and signaling mediator that coordinates a spectrum of oncogenic processes, including accelerated proliferation, migration, invasion, and the stabilization of the EMT [16,21,27,28,29]. A hallmark of TG2-mediated malignancy is its ability to engage diverse cell surface receptors, functioning as a multifunctional ligand within the tumor microenvironment [16,30]. Specifically, extracellular TG2 facilitates the assembly of ternary complexes with integrins and fibronectin to promote stable cell–matrix adhesion and survival signaling [19,23,30,31,32]. Furthermore, the adhesion G-protein-coupled receptor 56 GPR56 (ADGRG1) acts as a critical signaling receptor for extracellular TG2 [14,29,33]. In various aggressive cancers and cancer stem cell subpopulations, GPR56 is frequently hijacked and aberrantly overexpressed [33,34,35,36]. The interaction between these two proteins serves as a vital signaling nexus, whereby TG2 binding triggers receptor activation to drive the aggressive, stem-like phenotype of cSCC through downstream intracellular signaling cascades [14]. Unlike traditional GPCRs that primarily respond to soluble peptides or small molecules, GPR56 features a long extracellular region that heavily involves the receptor in cell-to-cell and cell–extracellular matrix (ECM) communication, making it a crucial regulator of tumor microenvironment dynamics [34,35,37].

This regulatory capacity makes the TG2-GPR56 axis a potent driver of malignancy not only in cSCC, but across a broad spectrum of solid tumors. TG2 and GPR56 are concomitantly co-expressed, and their cross-talk stimulates potent oncogenic signaling pathways (such as NF-κB) that accelerate tumor growth, invasion, and metastasis [34,38,39,40,41,42,43,44,45,46,47,48]. The functional consequences of this interaction are particularly well-illustrated in colorectal cancer (CRC), where TG2 promotes tumorigenicity by inactivating the tumor suppressor p53 [49]. Additionally, TG2-mediated cross-linking of CXCL12 and KRT19 proteins can suppress T-cell-mediated immune attack, contributing to immunotherapy resistance, a significant concern in advanced CRC [28,50]. Furthermore, functional knockdown studies of GPR56 have confirmed that silencing GPR56 reduces tumor growth, induces apoptosis via modulation of Bcl-2/Bax, and reverses EMT through the phosphoinositide 3-kinase/protein kinase B (PI3K/AKT) signaling [51]. The clinical relevance of GPR56 is further underscored by its correlation with increased metastatic potential, cellular proliferation, and invasive capacity [51,52,53,54]. Despite this well-documented oncogenic potential in cancer, the structural architecture of the TG2-GPR56 interaction, and how it specifically drives the aggressive, stem-like phenotype of cSCC, remain elusive.

**Figure 1:**
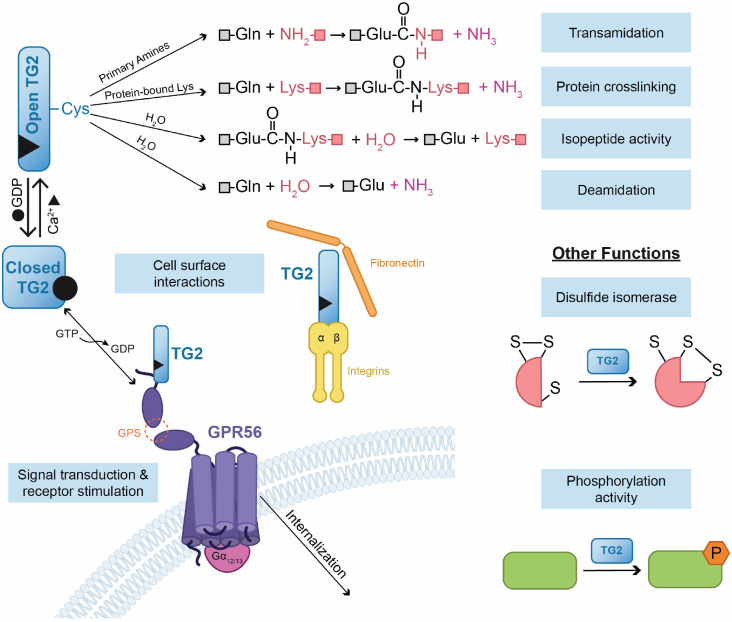
Enzymatic and non-enzymatic functions of TG2. TG2 catalyzes a Ca^2+^-dependent acyl-transfer reaction between the γ-carboxamide group of a specific protein-bound glutamine and either the ε-amino group of a distinct protein-bound lysine residue (covalent protein crosslinking; the principal in vivo activity) or primary amines such as polyamines and histamine. These reactions form polyamines or monoamine crosslinks with proteins or protein–protein crosslinks characterized by an ε-(γ-glutamyl)lysine isopeptide bond. Water can replace amine donor substrates, leading to the deamidation of the recognized glutamines. TG2 has other nonenzymatic functions that are based on its noncovalent interactions with multiple extracellular proteins, such as fibronectin and integrins. Emerging evidence highlights that TG2 GTPase activity plays a pivotal in regulation of transmembrane signaling through receptors like GPR56.

In this review, we highlight the dualistic and context-dependent roles of TG2 in cancer, identifying its interaction with the adhesion GPR56 as a potent, targetable pathway driving the aggressive phenotype of cSCC (Figure 1). Central to this discussion is the role of this molecular axis in ensuring the survival and maintenance of ECS cells, the subpopulation primarily responsible for treatment resistance and recurrence. While the definitive link between the TG2-GPR56 complex and systemic metastasis in cSCC continues to be elucidated, compelling evidence from related cancers and specific in vitro studies on keratinocytes provides a clear mechanistic roadmap. By dissecting these intricate molecular relationships, this work seeks to highlight the current understanding of the TG2-GPR56 interaction in malignancy, focusing on its non-canonical role translating extracellular cues into the activation of intracellular mechanism. An improved understanding of the structural biology of this intricate interaction would be essential for the design of next-generation anti-cancer therapies targeting this multifunctional protein.

## 2. TG2 Structure and Function

Tissue transglutaminase (also known as transglutaminase 2; TG2) is the most ubiquitously expressed member of the transglutaminase family (EC 2.3.2.13), a group of nine structurally and functionally related proteins comprising TG1 through TG7, blood coagulation factor XIII, and the catalytically inactive Band 4.2 [16,28,32]. Within the TG family, TG1, TG3 and TG5 are expressed in the epidermis where they are essential for forming the cornified envelope (CE) during keratinocyte differentiation from the suprabasal cell layers [16,55,56,57,58,59,60,61]. In contrast, TG2 is ubiquitously distributed, and plays distinct role. It is a unique, multifunctional oncogenic protein that has been intensely studied due to its role as a major cancer stem cell survival, inflammatory and metastasis factor. To drive these processes, TG2 stimulates multiple pro-cancer signaling cascades [21,23,62,63], in part, by interacting with various cell surface receptors [16,37,46,64]. Although predominantly a cytosolic protein, TG2 is also present in the nucleus and on the plasma membrane, where it displays GTPase, ATPase, protein kinase, and protein disulfide isomerase activity [16,65,66,67] (Figure 1). Pathologically, TG2 reduces expression of tumor suppressor genes, drives the synthesis and deposition of fibronectin and collagen to stabilize the extracellular matrix during tumor progression, and stimulates epithelial–mesenchymal transition (EMT) [1,62,68,69]. Consequently, TG2 levels and signaling activity are markedly increased in tumors [68,69,70,71,72], including epidermal cancer stem-like cells (ECS cells) where it stimulates the aggressive cancer phenotype [1,21,23,62,73].

**Figure 2:**
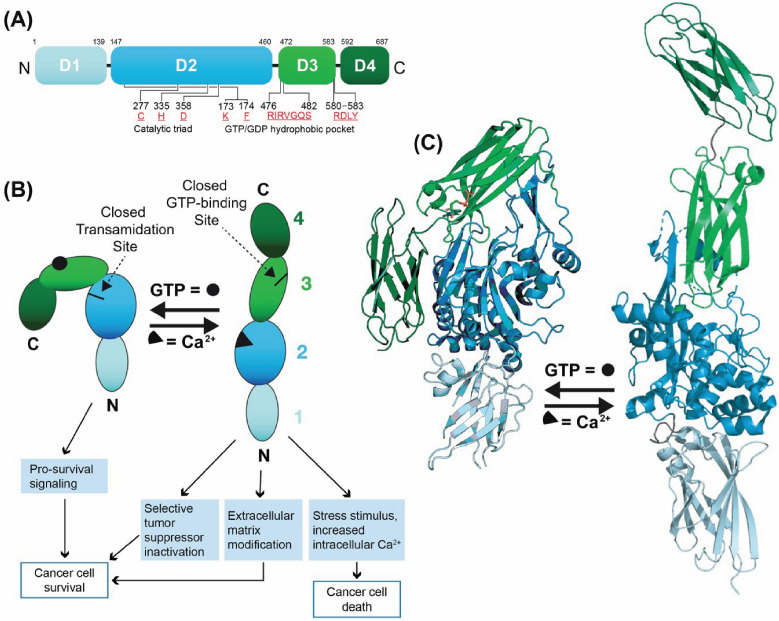
TG2 structural domain organization and function. (**A**) Domain structure organization: TG2 comprises an N-terminal β-sandwich domain (D1, cyan), a transamidase (TGase) domain (D2, blue), a GTP/GDP binding domain (D3, light green) and a C-terminal β-barrel domain (D4, green). (**B**) TG2 exists in two mutually exclusive, functional conformations. Intracellular GTP/GDP binding to the TG2 binding domain causes TG2 to assume a closed/folded/signaling-active GTP-bound conformation. In contrast, exposure to elevated free Ca^2+^ levels in the extracellular environment, or a stimulus-dependent increase in intracellular Ca^2+^, drives TG2 into an open/extended transamidase-active conformation. Notably, these functions are mutually restricted: GTP/GDP binding closes the transamidase domain, whereas Ca^2+^ binding closes the GTP/GDP-binding domain. (**C**) Cartoon representations of the open (PDB ID 9BC4) and closed (PDB ID 4PYG), colored according to panel (**A**).

Structurally, TG2 is composed of four domains, including an N-terminal β-sandwich, a catalytic core domain that contains the catalytic triad (Cys277, His335, and Asp358), and two COOH-terminal β-barrel domains (Figure 2). A distinctive feature of TG2 among the transglutaminase family is its GTP nucleotide-binding site, which is in a cleft between the catalytic core and the first β-barrel. This unique ability to bind GTP is strictly required for the stimulation of the aggressive cancer phenotype and cancer stem cell survival [16,21,32,63,74]. In response to high intracellular GTP levels, TG2 predominantly exists in a “closed,” pro-cancer signaling conformation. In this closed state, the two TG2 C-terminal β-barrel domains fold tightly over the central catalytic core, physically blocking substrates from accessing the catalytic triad [22,63,75,76,77,78,79]. However, when calcium level rise, Ca^2+^ binds to TG2, significantly reducing the enzyme’s affinity for GTP/GDP [16,28,63,80]. This binding causes the C-terminal β-barrel domains to swing away from the catalytic core. This shift into the open (extended) state exposes the active site, allowing TG2 to perform its transamidase (protein cross-linking) activity [16,63]. While TG2 contains multiple Ca^2+^-binding sites (at least five, designated S1 through S5), recent high-resolution structural studies show that the S1 and S3 sites are the primary drivers of TG2 open-state functionality (see Table 1 for human TG2 structures) [81]. In this open state, TG2 catalyzes Ca^2+^-dependent post-translational modifications of proteins through an acyl-transfer reaction (transamidation), introducing covalent bonds between free amine groups and the γ-carboxamide groups of peptide-bound glutamines to form stable ε-(γ-glutamyl) lysine cross-links (Figure 1). Thus, the closed conformation binds GTP and drives the cancer phenotype [63,75,76,77,78], while the open form catalyzes formation of intra- and interprotein ε-(γ-glutamyl) lysine bonds [65,82].

The balance between these open and closed states is tightly regulated by the distinct environments inside and outside the cell. In resting cells, calcium levels are extremely low (~100 nM) and GTP levels are high, meaning intracellular TG2 predominantly exists in the closed, catalytically inactive state. However, during extreme cellular stress or late-stage apoptosis, a massive influx of Ca^2+^ (reaching >700 nM or >1 mM) overrides the GTP, forcing TG2 into the open state to cross-link intracellular proteins [22,29,79,83]. Outside the cell, Ca^2+^ concentrations are high (in the millimolar range), which naturally drives TG2 into the open state. Moreover, the highly oxidative extracellular environment often causes the formation of the aforementioned disulfide bonds, keeping the open TG2 inactive until a reducing agent, such as thioredoxin, breaks the bonds to restore cross-linking activity [16,63]. In addition, TG2 catalyzes disulfide isomerase activities, phosphorylation and cell adhesion. These proteins serve as scaffolds, maintain membrane integrity, regulate cell adhesion, modulate signal transduction, and catalyze posttranslational modification of proteins via deamidation and amine incorporation (Figure 1) [14,16,63].

Given the profound impact of these multifaceted structural dynamics on cancer stem cell maintenance, it is clear why TG2 has emerged as a major therapeutic target. In preclinical models, the genetic ablation of TG2 reduces tumor size by up to 75%, while pharmacologic inhibition similarly produces a dramatic reduction in both tumor cell survival and overall tumor burden [27,28,62,84,85]. Because TG2 drives malignancy by binding to a host of membrane receptors to stimulate cancer cell survival, its targeted inhibition has been widely proposed as a potential anti-cancer strategy in humans [23,31,48,63,86,87]. However, although TG2 is recognized as a pivotal target for therapeutic intervention, comprehensive structural information regarding its interactions with cell receptors remains limited. Obtaining high-quality data on the multiple structural conformations of TG2 is therefore critical for the rational design and development of novel TG2-targeting drugs.

**Table 1:** Structures of human TG2 complexed with effectors and inhibitors.

Effectors/Inhibitors	Closed/Open	Method	PDB	References
GDP/Mg^2+^	Closed	X-ray	1KV3	[88]
GDP/Mg^2+^	Closed	CryoEM	8TR9	[89]
GTP/Mg^2+^	Closed	X-ray	4PYG	[78]
ATP/Mg^2+^	Closed	X-ray	3LY6	[90]
Ca^2+^	Open	X-ray	6KZB	[80]
Ca^2+^	Open	X-ray	9BC2	[81]
Ca^2+^	Intermediate	X-ray	9BC3, 9BC4	[81]
Ac-P(DON)LPF-NH_2_	Open	X-ray	2Q3Z	[75]
FNI2-3	Closed	Predicted model	9A3X	[91]
FNI7-9	Closed	Predicted model	9A3Y	[91]
FNI6FNII1-2FNI7-9	Closed	Predicted model	9A3Z	[91]
Inhibitor 1	Open	X-ray	3S3J	To be published
Inhibitor 2	Open	X-ray	3S3P	To be published
Inhibitor 3	Open	X-ray	3S3S	To be published

## 3. The Role of TG2 in Cancer

The pleiotropic nature of TG2 positions it as an essential, yet highly context-dependent, driver of the pathogenesis of numerous malignancies. While it is recognized as key multifunctional enzyme, its dysregulation in the tumor microenvironment directly fuels several defining hallmarks of cancer. TG2 is deeply implicated in cancer progression, often correlating with poor clinical outcomes, aggressive phenotypes, and metastasis across various cancer types [27,28,63,92,93,94]. Elevated TG2 levels have been documented in cancer cells and tissues including breast cancer [17,77,95,96,97,98,99,100,101,102,103,104,105,106,107], ovarian cancer [93,108,109,110,111,112,113,114], prostate cancer [115,116,117,118,119], lung cancer [120,121,122,123,124,125], leukemia [42,126], glioblastoma [67,127,128], renal cancer [129,130,131,132,133,134,135,136,137], epidermal squamous cell carcinoma [1,3,4,23,62,73,138,139,140,141,142,143,144], pancreatic cancer [62,73,145,146,147,148,149,150], cervical cancer [92,151], esophageal adenocarcinoma [34,152,153], oral squamous cell carcinoma [144,154,155,156,157], mesothelioma [13,158,159,160], gastric cancer [161,162,163,164,165] and colon cancer [162,163,166,167,168,169]. Furthermore, TG2 is strongly linked with the formation and maintenance of cancer stem cells (CSCs) by helping these cells acquire both stemness and plasticity, helping to maintain the cancer stem cell phenotype. To sustain this aggressive CSC phenotype and promote overall tumor metastasis, TG2 relies on a complex interplay of both its enzymatic (e.g., transamidase and GTPase) and non-enzymatic (e.g., scaffolding) functions (Figure 1). How TG2 deploys these specific functions is highly dependent on its subcellular location or its presence within the extracellular matrix. Because these multifaceted roles directly fuel metastatic spread and drug resistance, TG2 overexpression is widely considered a negative prognostic marker. Table 2 summarizes major mechanisms by which this complex protein promotes an oncogenic phenotype (search strategies are included in the Supplementary Information).

### 3.1. Cancer Stem Cell Survival and Maintenance

The aggressive clinical behavior of cSCC, particularly its propensity for recurrence, is heavily driven by a subpopulation of tumor cells known as epidermal cancer stem (ECS) cells. TG2 is intimately linked with the formation of this subpopulation, helping these cells acquire both stemness and plasticity. By operating through a non-canonical NF-κB pathway, TG2 actively increases the expression of CD44, a classic marker that promotes an aggressive cancer phenotype [24,161,174]. Unlike the rapidly dividing bulk of the tumor, ECS cells possess indefinite self-renewal capacity and are inherently refractory to cellular stress. The maintenance of this stem-like state is not exclusively hardwired into the genome; rather, it relies heavily on continuous signals from the tumor microenvironment (TME). TG2 is highly expressed in ECS cells and contributes to their survival and stemness [63]. TG2 knockdown or suppression of TG2 function with TG2 inhibitor (NC9) reduces ECS cell survival, spheroid formation, Matrigel invasion, migration, and epithelial-to-mesenchymal transition (EMT) [1,13,147]. Specifically, it is the GTP-binding/GTPase activity of TG2 that is responsible for maintaining the CSC phenotype and promoting cell survival [22,63], migration [108,137], and invasiveness [1,22,62]. This function is typically active when intracellular calcium levels are low, causing TG2 to adopt a closed, signaling conformation [78,80].

The mechanisms by which TG2 supports stem-like populations vary significantly across different cancer types. In cSCC, TG2 maintains ECS cell survival by interacting with α6/β4-integrin. This interaction stimulates FAK/Src signaling, which leads to PI3K activation and subsequently drives the YAP1/ΔNp63α signaling axis to maintain the cancer stem cell phenotype [23,62,63]. In melanoma, TG2 overexpression promotes cell survival and drug resistance by enhancing integrin mediated attachment [37,38,190]. Interestingly, TG2 and GPR56 exhibit antagonistic actions in melanoma: TG2 transamidase activity promotes melanoma cell growth, most likely by enhancing matrix deposition, while GPR56 binds to TG2 to inhibit this action and reduce fibronectin deposition [37,63]. Furthermore, low levels of TG2 reduce the nuclear translocation of the microphthalmia-associated transcription factor (MITF), supporting an undifferentiated and more invasive/mesenchymal phenotype akin to a stem-like state [191]. These contrasting mechanisms highlight the context-dependent nature of TG2, illustrating that while the closed, GTP-bound conformation drives stem cell survival and malignancy, its open, Ca^2+^-activated transamidase cross-linking activity can conversely trigger the cell death process.

### 3.2. Chemo- and Radiation-Resistance

The ability of TG2 to inherently shield cancer stem cells from apoptosis underscores its role in driving mechanisms of acquired therapeutic resistance across various tumor types. TG2 confers resistance to standard cancer therapies through activation of different oncogenic pathways, notably the NF-κB survival pathway, which is a key anti-apoptotic mechanism. For instance, the transamidase function of TG2 can crosslink IκBα (an inhibitor of NF-κB), leading to its proteasomal degradation and subsequent constitutive activation NF-κB. Moreover, TG2 initiates “outside–in” survival signaling by regulating the cellular adhesion to the extracellular matrix, which activates pathways like the focal adhesion kinase (FAK) and the AKT/β-catenin cascades [16,30,192]. Additionally, some studies demonstrate that TG2 physically interacts with the tumor suppressor p53. This interaction facilitates the transport of p53 to autophagosomes, leading to its degradation. By promoting p53 depletion, TG2 disables the cell’s apoptotic machinery and significantly increases tumor cell survival and drug resistance [83,132,193].

In cSCC, TG2-driven signaling networks heavily skew the intracellular apoptotic rheostat. By upregulating anti-apoptotic proteins like Bcl-2 and shifting the Bcl-2/Bax ratio, TG2 raises the apoptotic threshold against severe DNA damage induced by chemoradiation. Furthermore, extracellular TG2 facilitates resistance to targeted EGFR inhibitors via a unique mechanism, the binding to receptors like GPR56 to activate a disintegrin and metalloproteinase 17 (ADAM17). This TG2-mediated ADAM17 activation leads to the shedding of EGFR ligands, which transactivates EGFR independently of the targeted therapeutic blockade to drive survival and cellular migration [14]. In breast cancer, TG2 promotes resistance to a broad array of common anti-cancer drugs, including doxorubicin, docetaxel, histone deacetylase (HDAC) inhibitors such as vorinostat, and mTOR inhibitors like rapamycin [19,102,103]. The mechanisms driving this resistance are highly adaptable. For example, TG2 mediates resistance to the targeted kinase inhibitor neratinib by upregulating interleukin-6 (IL-6) and inducing the JAK/STAT3 pathway [28]. Additionally, TG2 acts as a predictive marker and driver for resistance to immunotherapy (PD-L1 inhibitors). In this context, TG2 induces the proteasomal degradation of PTEN and IκBα, hyperactivating the PI3K/AKT and NF-κB signaling pathways to upregulate PD-L1 and the chemokine CCL2, which effectively restricts T-cell motility and drives immune evasion [17,28]. In other malignancies, such as melanoma and osteosarcoma, high TG2 expression correlates with chemotherapy resistance. In melanoma, cells with lower TG2 expression demonstrate increased sensitivity to the drug dacarbazine [28]. In these resistant cells, TG2 activates the integrin signaling pathways to directly enhance the cell’s survival advantage against cytotoxic stress [28,63]. Similarly, elevated TG2 in osteosarcoma protects cells from apoptosis under hypoxic conditions by suppressing Bax and reducing cytochrome c release, a mechanism directly associated with driving resistance to the chemotherapeutic drug cisplatin [17,194].

### 3.3. Metastasis and Invasion

Metastasis and invasion represent the most lethal aspects of cancer progression, driven by the tumor’s ability to disseminate, survive detachment, and infiltrate secondary sites. TG2 acts as a strong promoter of metastasis, primarily by inducing EMT, a critical process where epithelial cancer cells lose their polarity and cell-to-cell adhesion, gaining the migratory and invasive properties typical of mesenchymal cells. Furthermore, the ability of TG2 to drive the acquisition of stem cell-like phenotypes and regulate the extracellular matrix (ECM) heavily promotes tumor metastasis and drug resistance [17,28,63].

In cSCC, recent evidence indicates that extracellular TG2 fundamentally drives epithelial motility through its interaction with GPR56, rather than relying solely on enhanced matrix tension. TG2 directly binds to the N-terminal domain of GPR56, which initiates an intracellular signaling cascade involving the RhoA-associated protein kinase (ROCK) pathway. This activation mechanism is known to regulate cellular migration in keratinocytes and other cells through ROCK pathway. Crucially, this TG2-GPR56 signaling axis directly activates the metalloproteinase ADAM17. Because GPR56 is overexpressed in aggressive cSCC, the acquisition of an invasive phenotype is closely linked to this increased ADAM17-mediated shedding of EGFR ligands and subsequent transactivation of EGFR [14]. Concurrently, downstream signaling upregulates mesenchymal transcription factors (such as Snail and Twist) and represses E-cadherin, allowing individual tumor cells to physically detach and migrate through the dense ECM into surrounding tissues and lymphatic vessels in vivo [17,30]. In this context of metastasis, perineural invasion (PNI) is a high-risk feature that carries a significant risk for locoregional and distant spread [11].

At the plasma membrane, the interaction of TG2 with integrins and fibronectin is essential for activating “outside–in” signaling pathways, such as FAK, PI3K/Akt, β-catenin, and Src, which are critical for cell survival, migration, and metastasis [30]. In the extracellular space, high Ca^2+^ levels activate TG2 transamidase function to cross-link matrix proteins (like collagen and fibronectin), altering ECM stiffness and influencing cell adhesion, motility, and invasion. Intracellularly, the transamidation of the GTPase RhoA by TG2 can render it constitutively active, further driving cell motility and invasion [141,195]. In breast cancer, TG2 drives EMT by activating NF-κB, which increases the expression of mesenchymal transcription factors like Snail1, Zeb1, Zeb2, and Twist1, while causing the loss of E-cadherin. Extracellularly, TG2 cross-links the S100A4 protein into multimers that bind syndecan-4, activating α5β1-integrin “outside–in” signaling to stimulate tumor cell migration. Furthermore, weakly migratory metastatic cells release microvesicles containing TG2; these microvesicles activate lung fibroblasts and induce fibronectin fibrillogenesis and matrix stiffening, which facilitates tumor spreading and the establishment of a pulmonary metastatic niche [28,63,191].

## 4. TG2 Is Highly Expressed and Required in cSCC Stem Cells

As established in prevous sections, TG2 expression is markedly elevated in cSCC and is strictly required for epidermal cancer stem-like (ECS) cell subpopulation, where its closed-conformation GTP-binding activity is strictly required for survival and sphoroid formation [1,21]. Additionally, extracellular TG2 serves as a critical scaffolding ligand for diverse membrane receptors, including integrins [23,31,87] and GPR56 [48,196], to stimulate “outside–in” oncogenic signaling, EMT, and metastatic dissemination. Because suppressing TG2 function dramatically reduces ECS survival and triggers apoptosis, the TG2–receptor signaling axis is widely recognized as a highly attractive therapeutic target. Despite this recognition, limited structural insight into how TG2 dynamically engages its wide array of membrane targets has severely hindered the understanding of these specific oncogenic signaling processes and slowed the rational development of new inhibitors. While certain active site-directed inhibitors (such as NC9, VA4, and VA5) have been shown to shift TG2 conformation from a closed to an open state, as measured by FRET/FLIM spectroscopy, the precise molecular details of these structural changes remain incompletely resolved [22,139,197].

At the plasma membrane, the structural interaction of TG2 with integrins and fibronectin is essential for activating “outside–in” signaling pathways, such as FAK, PI3K/Akt, β-catenin, and Src—which are critical for cell survival, migration, and metastasis [23,30,31,87]. Mechanistically, the TG2 N-terminal β-sandwich domain binds with high affinity to the 42 kDa gelatin-binding domain of fibronectin, acting as a stable bridge to cell surface β-integrins [30,32,192]. This fibronectin–TG2–integrin ternary complex enhances integrin clustering and focal adhesion stability. This structural engagement activates “outside–in” signaling cascades, involving kinases such as FAK, Src, and RhoA, that are essential for the EMT, the maintenance of cancer stemness, and metastatic dissemination. Surface accumulation of TG2 can occur via the translocation of intracellular TG2 or through the binding of secreted extracellular TG2 back to the cell surface, where it serves as a ligand for diverse membrane receptors, including GPR56 [31,91,198]. However, while the biological consequences of these interactions are increasingly clear, the specific structural mechanisms that govern how both intracellular and extracellular TG2 dynamically bind to these cell surface receptors to stimulate the aggressive cancer phenotype remain to be fully defined.

## 5. GPR56: A Key Receptor in Neurological Disorders and Cancer

**Figure 3:**
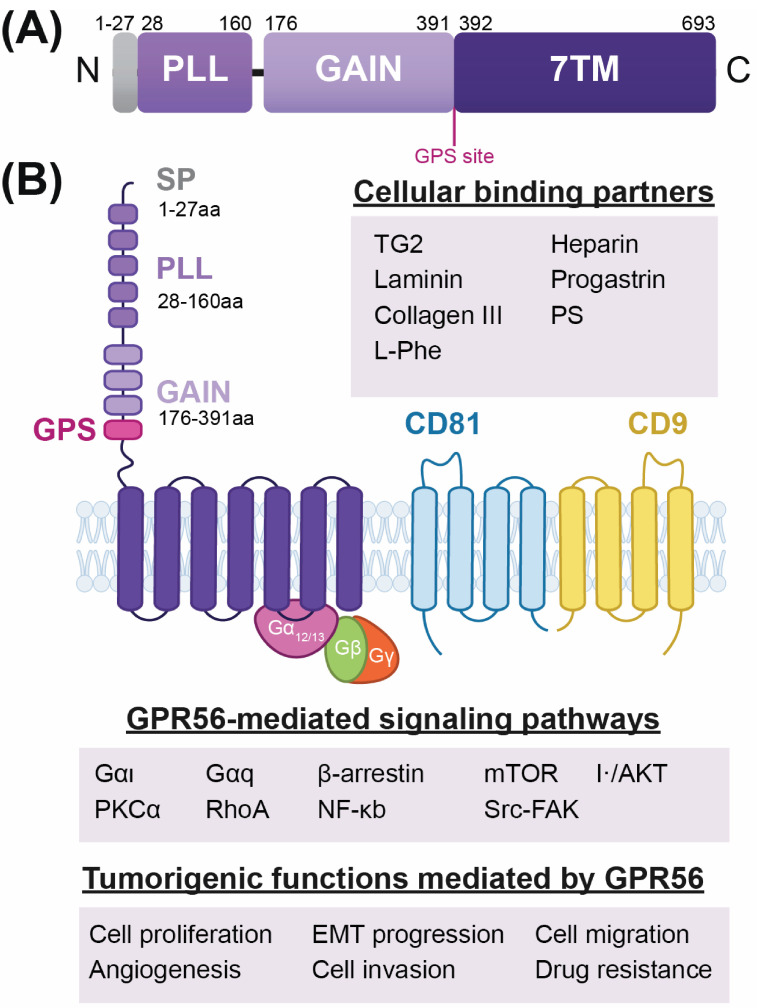
Overview of the ADGRG1/GPR56 protein. (**A**) GPR56 sequential domains. The signal peptide (SP, residues 1–27) is depicted in gray. The pentraxin/laminin/neurexin/sex hormone-binding globulin-like (PLL) (residues 28–160) and the G-protein-coupled receptor autoproteolysis inducing (GAIN) (residues 176–391) domains are depicted in purple. I The seven-pass transmembrane domain (7TM) is depicted in dark purple. The GPS, GPCR proteolysis site, is indicated in magenta. (**B**) Upper panel: schematic depiction of GPR56 receptor domain organization. Domain organization: (1) the GPR56 N-terminal fragment (NTF) is composed of the GAIN and PLL domains, as well as the GPS proteolysis site; the (2) GPR56 C-terminal fragment (CTF) includes the seven-pass transmembrane (7TM domain responsible for associating with and activating intracellular G-proteins, primarily Gα_12/13_ and Gq. Lower panel: GPR56 cellular ligands and binding partners (i.e., TG2, laminin, collagen III, L-Phe heparin, progastrin, PS), GPR56-mediated signaling pathways (i.e., CD81 and CD9), and tumorigenic functions are underlined in purple boxes, as published [36,48].

The adhesion G-protein-coupled receptors (aGPCRs) constitute a large family of membrane proteins. Members of the aGPCR family contain extracellular regions (ECRs) that participate in cellular communication to regulate cell size, shape, polarity, adhesion, migration, cycle, death, and differentiation. GPR56 (also known as ADGRG1) is a highly versatile adhesion aGPCR that plays critical roles in brain development [36,199], tumorigenesis, and cancer progression [14,33,38,39,45,46]. GPR56 is expressed in many ectodermally derived cells as a single polypeptide but undergoes intracellular autoproteolysis during maturation in the endoplasmic reticulum. This self-cleavage is catalyzed by the highly conserved GPCR autoproteolysis-inducing (GAIN) domain at a specific GPCR proteolysis site (GPS). This results in two distinct set of domains that remain non-covalently attached after presentation at the cell surface: (1) the extracellular N-terminal fragment (NTF), which comprises the N-terminal portion of the GAIN domain; and (2) the C-terminal fragment (CTF), which includes the C-terminal portion of the GAIN domain (containing the encrypted tethered agonist or Stachel sequence), a seven-pass transmembrane (7TM) domain, and an intracellular domain (ICD) that is responsible for associating with and activating intracellular G-proteins, primarily Gα_12/13_ and G_q_ (Figure 3) [38,48,64,196,200,201].

GPR56 functions as a molecular switch to activate various intracellular signaling pathways; indeed, multiple studies have shown that GPR56 overexpression activates various signaling pathway response elements (Table 3). While its activation mechanisms are multifaceted, a tethered agonism model is widely accepted. In this model, canonical activation occurs when extracellular ligands, such as TG2, collagen III, or laminin, bind to the PLL domain. This engagement causes a structural change or triggers the dissociation of the NTF-CTF complex, which exposes the tethered agonist, driving structural 7TM rearrangements that lead to downstream G_α12/13_-dependent signaling [37,38,46,48,64,196]. Recent high-resolution cryo-EM structural investigations of GPR56 and latrophilin-3 (LPHN3), in complex with a G_13_ protein variant, have elucidated this process in detail [202]. The study describes the switch between the inactive/NTF-bound state holoreceptor, where the NTF remains non-covalently attached to the CTF, and the active/G-protein-coupled state. Upon exposure, the tethered agonist engages the 7TM core, stabilizing a fully active receptor conformation that enables G-protein coupling and consistent RhoA activation [14,46,202]. Mechanistically, these cellular processes are fundamentally driven by GPR56 coupling to intracellular G-proteins—primarily Gα_12/13_ and Gq—leading to consistent RhoA activation. In this context, TG2 and collagen III have been identified as its primary functional ligands [200,201,203]. When this normal physiological ligand–receptor interaction is hijacked within the tumor microenvironment, it becomes a potent driver of malignancy. Therefore, GPR56 plays a key role in cell proliferation, migration, adhesion, and survival.

These sources examine the complex roles of TG2 and the adhesion GPR56 in cancer progression and mortality. Research indicates that TG2 acts as a multifunctional enzyme that influences tumor microenvironments, promoting epithelial–mesenchymal transition and cancer stem cell survival in various malignancies. Studies on GPR56 reveal its necessity for bone metastasis and its direct interaction with TG2, which can trigger receptor activation through structural changes. Additionally, population-based data highlight increased mortality risks for patients with a history of cutaneous squamous cell carcinoma, particularly among organ transplant recipients. Scientists are also developing synthetic ligands and small-molecule inhibitors to modulate these proteins, aiming to suppress cancer cell invasion and improve patient outcomes. Together, these findings map the molecular mechanisms and epidemiological trends that define aggressive cancer behavior [28,48,196].

## 6. The Role of TG2 and GPR56 Interaction in Cancer Invasion

**Figure 4:**
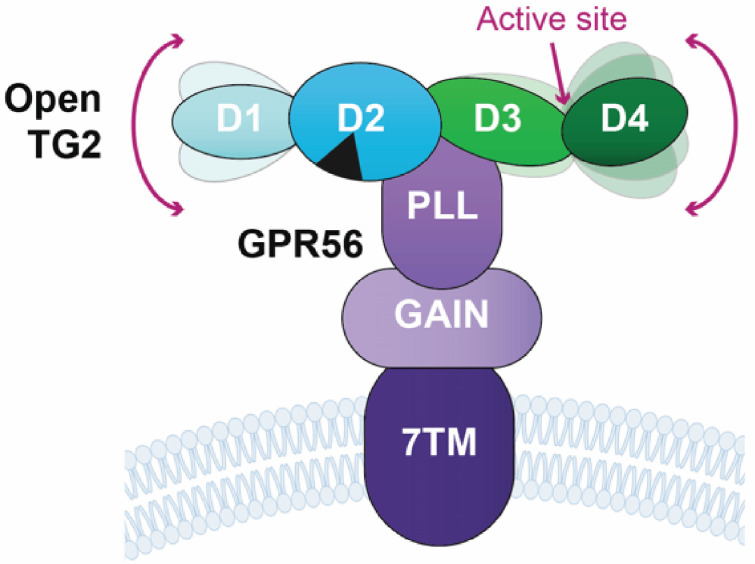
TG2 proposed interaction model with the GPR56 receptor. Schematic representation of in open conformation TG2 interacting with the GPR56 receptor. TG2 is composed of four domains: an N-terminal β-sandwich domain (D1; aa 1–139), a transamidase (TGase) domain (D2; aa 139–470), a GTP/GDP binding β-barrel domain (D3; aa 471–586), and a C-terminal β-barrel domain (D4; aa 587–688). The GPR56 receptor is anchored to the cell membrane via its transmembrane domain (7TM) and exposes its N-terminal extracellular region, composed of the GAIN and PLL domains. The model proposes that the TG2 β-barrel domain (D3) binds directly to the PLL domain of the GPR56 receptor to activate cellular signaling [48,196]. The arrows indicate TG2 active, calcium bound, open conformation as a key functional state for binding to GPR56.

The interaction between TG2 and GPR56 plays a highly significant role in regulating cancer invasion, particularly in melanoma [14,37,38,46,63]. This conformation-dependent interaction is specifically mediated by the C-terminal β-barrel domains of TG2 binding to a highly conserved, surface-exposed patch on the PLL domain of the receptor (Figure 4) [46,48,196]. The interaction relies heavily on critical PLL residues such as H89, but notably does not require the enzymatic catalytic activity of TG2. Mechanistically, the binding of TG2, sometimes requiring an extracellular matrix co-factor like laminin to form a functional tripartite complex, induces a conformational change that triggers the dissociation or “shedding” of the NTF. Upon NTF dissociation, the Stachel peptide agonist becomes exposed, inserting itself into the 7TM core to stabilize the receptor in a fully active conformation. Once active, the 7TM domain primarily couples to intracellular heterotrimeric G-proteins, predominantly G_α12/13_, which subsequently drives downstream RhoA signaling. Given TG2’s well-established role as a major driver of cancer stem cell survival, inflammation, and metastasis, this receptor–ligand pairing is of high clinical interest.

The functional outcomes of the TG2-GPR56 complex are highly context-dependent; while it drives malignancy in most carcinomas [14,34,208], in melanoma, the two proteins exhibit an antagonistic relationship. When acting independently, extracellular TG2 cross-links the matrix to enhance cell–matrix adhesion and melanoma growth. However, GPR56 functions as a critical tumor suppressor in this environment and is essential for limiting melanoma progression through two primary, receptor-mediated mechanisms: (1) Internalization and degradation of TG2: GPR56 directly antagonizes the tumorigenic and pro-invasive effects of TG2 by acting as a clearance receptor. Upon binding TG2 at the cell surface, GPR56 rapidly internalizes the enzyme and targets it for degradation within lysosomes. This receptor-mediated clearance reduces TG2 accumulation in the extracellular matrix, effectively preventing excessive matrix cross-linking, impairing focal adhesion kinase (FAK) signaling, and limiting cancer growth, metastasis, and invasion [37]. (2) Suppression of tumor angiogenesis: The TG2-GPR56 interaction also triggers an intracellular signaling cascade that suppresses tumor angiogenesis, a critical step for metastatic dissemination. Specifically, this receptor–ligand engagement inhibits the production of vascular endothelial growth factor (VEGF) via a protein kinase C alpha (PKCα)-mediated signaling pathway [36,209]. However, while this tumor-suppressive clearance mechanism is critical in melanoma; in most other malignancies—such as cSCC and aggressive glioblastomas—GPR56 is highly overexpressed and hijacked to drive tumor progression [14,36].

Recent studies have underscored that the acquisition of an aggressive cancer phenotype, specifically characterized by enhanced tumor motility and the ability to invade, can be explained by the localized, TG2-driven, ADAM17-mediated transactivation of EGFR in these tumors [14]. On the surface of malignant keratinocytes, extracellular TG2 binds to the N-terminus of GPR56, which subsequently activates ADAM17. This membrane-bound metalloprotease then cleaves and sheds membrane-bound EGFR ligands (such as TGF-α and amphiregulin). The resulting shedding triggers EGFR transactivation, driving rapid keratinocyte migration and invasive responses. While no ADAM17-specific inhibitor is currently FDA-approved for cSCC, the TG2-driven activation of ADAM17, and the resulting cell migration and proliferation, can be effectively targeted using specific pharmacological inhibitors [14]. Interrupting this signaling cascade, using specific ADAM17 inhibitors, completely blocks the shedding of EGFR ligands, halting both TG2-mediated cell migration and proliferation. Additionally, broad-spectrum metalloproteinase inhibitors like GM6001 and TAPI-1 can partially inhibit TG2-driven migration. Furthermore, applying a specific ROCK inhibitor (such as Y-27632) successfully blocks ADAM17 from releasing EGFR ligands in both TG2-stimulated and non-stimulated cells. In experimental models, treatment with the specific EGFR kinase inhibitor AG1478 completely blocks cell migration driven by extracellular TG2 [14]. Clinically, this mechanism is highly relevant, as currently utilized therapies include a combination of inhibitory monoclonal antibodies (i.e., cetuximab) designed to specifically bind to and inhibit the EGFR signaling pathway.

The TG2-GPR56 interaction has been studied in different experimental settings [38,46,47,48]. The development of novel therapeutics targeting TG2-GPR56 interactions requires the solution of the TG2-GPR56 complex structure. Since no experimental structure for human TG2-GPR56 complex is available, recent studies have investigated the biochemical and structural basis for the TG2 and GPR56 interaction by carrying out a structure-guided mutagenesis to pinpoint the binding interface on the PLL domain of GPR56 [48,196]. This interaction specifically requires the C-terminal domain of TG2. Additionally, researchers have generated monoclonal antibodies directed against the NTF of GPR56 [196], which is the specific domain where TG2 binds [48], used as tools to understand GPR56’s structure and to reveal its intracellular signaling pathways, such as its regulation of cell migration via RhoA [36]. Rather than blocking the pathway, several functional monoclonal and polyclonal antibodies (such as N-GPR56-Fc-fusion protein) have been designed to act as agonists. The development and use of these monobodies, along with other specific antibodies and small-molecule interactors, have proven highly valuable as experimental tools. By acting as targeted agonists or antagonists, they allow researchers to delineate the complex intracellular signaling cascades controlled by GPR56. Experimental studies demonstrate that these monobodies can effectively block TG2 from interacting with the conserved binding residues on the N-terminal domain of GPR56 [14].

## 7. Conclusions and Future Directions

This review supports several important conclusions. First, TG2 is a key cancer cell survival factor that triggers various signaling mechanisms to drive epithelial–mesenchymal transition (EMT), cancer stem cell survival, angiogenesis, inflammation, and metastasis. Moreover, TG2 levels are elevated across a broad spectrum of malignancies, where this overexpression is associated with advanced disease, metastasis, disease recurrence, poor prognosis, and reduced patient survival. Second, TG2 conformation is a key determinant of its pro-cancer activity. The GTP-bound (closed/signaling-active) conformation of TG2, which predominates in the intracellular environment, is a major driver of cancer cell survival. In contrast, the calcium-bound (open/transamidase-active), in the extracellular space, is well known for cross-linking proteins (i.e., fibronectin and collagen) and acting as a protein scaffold by binding to integrins and other cell receptors, such as GPR56. Through these interactions, extracellular TG2 triggers intracellular signaling cascades (like FAK, Src, and NF-κB), which are implicated in promoting cancer cell survival, migration, invasion, and EMT. Ultimately, the GTP-bound form appears to be the predominant driver of cancer cells [16,21,27,28,29,63].

TG2 has been successfully validated as a druggable clinical target, most notably by ZED1227, a highly specific small-molecule inhibitor that forms a stable covalent bond with the catalytic center of TG2. While ZED1227 is currently undergoing clinical evaluation primarily for celiac disease, its clinical safety profile provides strong proof-of-concept for deploying TG2 inhibitors in human patients. Meanwhile, the development of dual-action TG2 inhibitors, which inhibit both TG2-GTP binding and transamidase activity, remains predominantly at the preclinical stage. Small molecules such as NC9 and VA4 covalently bind to the TG2 catalytic site, abolishing its dynamic conformational shifts and GTPase activity. By locking TG2 in an extended open conformation, these agents indirectly distort the GTP-binding pocket, thereby abolishing GTPase signaling activity. Consequently, VA4 has recently demonstrated the ability to prolong survival in preclinical xenograft models of ovarian cancer [210,211].

Because TG2 pro-cancer activity is heavily dictated by its structural state, newer therapeutic strategies focus on compounds designed to stabilize specific conformations. In this context, TTGM-5826 acts as a competitive inhibitor that stabilizes the “open” form of TG2, effectively promoting cytotoxicity and inhibiting the growth and migration of cancer cells with high TG2 expression [22]. Conversely, molecules such as LDN-27219 have been reported to stabilize the “closed” conformation of TG2, effectively inhibiting tumor progression in in vivo colorectal cancer models. Another strategy relies on treatment with extracellular agents that increase intracellular calcium levels, forcing the activation of TG2 transamidase activity and shifting the enzyme into a cytotoxic state to kill cancer cells [50,79].

Finally, targeting the downstream receptor GPR56 with traditional small molecules has historically been challenging. While certain small molecules, like dihydromunduletone (DHM), have been identified as selective GPR56 antagonists, novel biologics are now rapidly advancing. Recent developments have introduced antibody–drug conjugates (ADCs) directed against GPR56. For instance, a novel ADC utilizing the anti-GPR56 antibody 10C7—which binds proximal to the receptor’s autoproteolysis site (at residue H360) and rapidly co-internalizes to the lysosome—has demonstrated significant efficacy in preclinical models of colorectal cancer [212,213]. Importantly, biochemical studies have now confirmed that GPR56 specifically binds to the Ca^2+^-bound TG2 (open conformation). This direct, high-affinity interaction is explicitly mediated by the C-terminal β-barrel domains (D3 and D4) of TG2, which bind to a highly conserved, surface-exposed patch on the PLL domain of GPR56 [48]. Furthermore, landmark cryo-EM visualizations have recently captured GPR56 in its fully active, G-protein-coupled state bound intramolecularly to its native tethered agonist, elucidating the precise structural rearrangements required for receptor activation [202]. With these high-resolution structural frameworks now established, future therapeutic development can focus on utilizing synthetic ligands, such as targeted monobodies, to competitively block the specific TG2-GPR56 binding interface, paving the way for the rational design of next-generation targeted therapies.

## Figures and Tables

**Table 2:** TG2 and hallmarks of cancer.

Cancer Impact	Key Pathways and Mechanisms	References
Stimulation of cell proliferation and aggressive cancer phenotype	β-catenin/Wnt, TGF-β, PI3K/AKT, CD44v6/ERK1/2 Signaling	[24,25,158,170,171]
Increased cell invasiveness, survival and metastasis	EMT-CSCs, β-catenin and VEGF, MVP mediated MAPK/ERK1/2 signaling	[20,63,69,77,172,173,174,175,176,177]
Survival and treatment resistance	ECS, TRAIL, Caspase-3/Bax	[49,178,179,180,181]
Evading growth suppressors	RB/p53 pathway regulation	[50,96,128,182,183]
Inducing angiogenesis	NF-κB/HIF1α, VEGF, ECM remodeling	[20,184,185,186,187,188,189]
Contribution to TME and tumorigenesis	EGF, TGF-β induced EMT, Rac	[67,108,173,174]

Abbreviations: RB: retinoblastoma protein; TGF-β: transforming growth factor beta; PI3K: phosphoinositide 3-kinase; TRAIL: tumor necrosis factor-related apoptosis-inducing ligand; NF-κB: nuclear factor kappa B; HIF1α: hypoxia-inducible factor1α; VEGF: vascular endothelial growth factor; ECM: extracellular matrix; EMT: epithelial–mesenchymal transition; EGF: endothelial growth factor; TME: tumor microenvironment.

**Table 3:** GPR56 signaling pathways.

Ligands/Activators	GPR56 Domain	Downstream Signaling Pathway	References
Collagen III	CTF/NTF	RhoA-ROCK-MLC/JAK-STAT3	[204,205]
Transglutaminase 2 (TG2)	NTF	MES transition/NF-kB	[46]
Transglutaminase 2 (TG2)	CTF	ECR-PLL domain	[48,196]
Epidermal growth factor (EGF) receptor	NTF	RhoA-ROCK-ADAM17	[14]
Androgen receptor (AR)	Gas/	RhoA-cAMP/PKA	[206]
Tetraspanin complexes (CD9/CD81)	Gq/Gα_12/13_	RhoA, β-arrestin recruitment	[45,205]
Heparin	Gα_12/13_	Rho signaling	[45,207]
Biased signaling	Gα_12/13_	JAK-STAT3 pathway	[204]

## Data Availability

No new data were created or analyzed in this study. Data sharing is not applicable to this article.

## Supplementary Materials

The following supporting information can be downloaded at: https://www.mdpi.com/article/10.3390/ijms27062902/s1.

## Abbreviations

The following abbreviations are used in this manuscript:

ASPR	Age-standardized prevalence rate
ASIR	Age-standardized incidence rate
TG1	Transglutaminase 1
TG2	Transglutaminase 2
TG3	Transglutaminase 3
TG5	Transglutaminase 5
CE	Cornified envelope
GTP	Guanosine triphosphate
cSCC	Cutaneous squamous cell carcinoma
aGPCRs	Adhesion G-protein-coupled receptors
GPR56	G-protein-coupled receptor 56
ADGRG1	Adhesion G-protein-coupled receptor G1
NTF	N-terminal fragment of GPR56
CTF	C-terminal fragment of GPR56
PLL	Pentraxin, Laminin, neurexin, and sex hormone-binding globulin-Like domain
GAIN	G-protein-coupled receptor Autoproteolysis INducing domain
GPS	G-protein-coupled receptor proteolysis site
7TM	Seven-pass transmembrane domain
ECS	Epidermal cancer stem
EMT	Epithelial–Mesenchymal Transition
UI	Uncertainty Interval
ECM	Extracellular matrix
CRC	Colorectal cancer
BCL-2	B-cell lymphoma 2 anti-apoptotic protein
PI3K/AKT	Phosphoinositide 3-kinase (PI3K)/Protein kinase B (AKT)
CSC	Cancer stem cell
NF-κB	Nuclear factor kappa-light-chain-enhancer of activated B-cells
Wnt	Wingless-related integration site
TGF-β	Transforming growth factor-β
CD44v6	Cluster of differentiation 44 variant 6
EGFR	Epidermal growth factor receptor
Src	Proto-oncogene tyrosine-protein kinase Src
MITF	Microphthalmia-associated transcription factor
YAP1	Yes-associated protein 1
ΔNp63α	Delta Np63 alpha, p53-family transcription factor
ROCK	RhoA-associated protein kinase
LPHN3	Latrophilin-3
ADGRL3	GPR56 and latrophilin-3
ADAM17	Metalloproteinase 17
VEGF	Vascular Endothelial Growth Factor
CXCL12	CXC motif chemokine 12, also known as stromal cell-derived factor 1 (SDF-1)
KRT19	Keratin 19
CD81	Cluster of Differentiation 81
CD9	Tetraspanin CD9 protein
