# Supplementary material for: The Role of the TG2-GPR56 Complex in Cutaneous Squamous Cell Carcinoma (CSCC) Aggression and Therapeutic Resistance
Source: Int J Mol Sci. 2026 Mar 23;27(6):2902. doi: 10.3390/ijms27062902 (PMC13026756; doi:10.3390/ijms27062902)
Supplement: Supplementary file 1 [file ijms-27-02902-s001.zip › ijms-4148377-supplementary.pdf]

Supplementary Information

This review is conceived as a comprehensive narrative synthesis. The total number of records initially retrieved was 380. However, after removal of duplicates and application of the inclusion and exclusion criteria, the final number of studies included in this review was 213. To ensure maximum coverage of the fundamental and contemporary literature, the following keywords were used in the literature search:

"Transglutaminase 2" OR "TG2"
"GPR56" OR "ADGRG1"
"TG2–GPR56 interaction" OR "TG2–GPR56 complex"
"Cutaneous squamous cell carcinoma" OR "cSCC"
"Epidermal cancer stem cells" OR "ECS cells"
"Epithelial-mesenchymal transition" OR "EMT"
"Cancer stem cells" OR "CSC"
"ADAM17" AND "EGFR transactivation"
"Tumor invasion" OR "cancer invasion"
"Cell survival" OR "tumor sphere" OR "spheroid"
"TG2 inhibitors" OR "transglutaminase 2 inhibitors"

In addition, we used the following Boolean operators across search databases:

((Transglutaminase 2) OR (TG2)) AND (cancer)
AND
((Transglutaminase 2) OR (TG2)) AND (cell carcinoma)
AND
((Transglutaminase 2) OR (TG2)) AND (squamous cell carcinoma)
((Transglutaminase 2) OR (TG2)) AND (squamous cell carcinoma) AND inhibitor)
((Transglutaminase 2) OR (TG2)) AND ((squamous cell carcinoma))
OR
((Transglutaminase 2) OR (TG2)) AND ((cancer stem cells) OR (ECS))
OR
((Transglutaminase 2) OR (TG2)) AND ((epithelial mesenchymal transition) OR (EMT))
OR
((Transglutaminase 2) OR (TG2)) AND ((invasion) AND (cell survival))
((GPR56 OR ADGRG1) AND (cancer OR tumorigenesis OR carcinoma)
OR
((GPR56 OR ADGRG1) AND ((squamous cell carcinoma) OR SCC OR carcinoma)
((GPR56 OR ADGRG1) AND ((Transglutaminase 2) OR (TG2))
OR
((GPR56 OR ADGRG1) AND ((Transglutaminase 2) OR (TG2)) AND complex)
OR
((Transglutaminase 2) OR (TG2)) AND (ADAM17 OR EGFR))
((Transglutaminase 2) OR (TG2)) AND GPR56 AND (treatment OR therapy))
OR
((Transglutaminase 2) OR (TG2)) AND GPR56 AND (treatment OR therapy) AND Inhibitor)

Additional refinement terms used when needed:

"ADAM17" AND "EGFR shedding"
"TG2 open conformation" AND "Ca2+"
"GPCR adhesion receptor" AND "tethered agonist"
"TG2 GTP-binding activity"

"TG2 inhibitors" OR "NC9" OR "VA4"

Inclusion and exclusion criteria applied during the screening process:

Inclusion criteria: original research articles and review papers addressing TG2 biology, GPR56 biology, TG2–GPR56 interactions or complexes, cSCC, cancer stem cells, EMT, therapeutic resistance, or invasion; studies involving human samples, animal models, or in vitro systems; and articles available in English.

Exclusion criteria: non-peer-reviewed sources (e.g., conference abstracts, preprints); studies unrelated to TG2 or GPR56 despite containing overlapping keywords; and articles unrelated to cancer biology or cutaneous malignancies.
